# Dose-dependent mortality risk of plasma transfusion in critically ill patients

**DOI:** 10.1016/j.htct.2026.106491

**Published:** 2026-06-23

**Authors:** Min Zhang, Hongjun Gao, Chushu Liao, Fen Yuan, Zhen Huang, Xisha Huan, Shan Feng, Fangfang Chen, Ping Lei

**Affiliations:** a Department of Transfusion Medicine, Hunan Provincial People’s Hospital (The First Affiliated Hospital of Hunan Normal University), Hunan, China; b Blood Transfusion Institute, Jiangsu Zojiwat Biopharmaceutical Co., Ltd., Wuxi, Jiangsu, China

**Keywords:** Plasma transfusion, Frozen plasma, In-hospital mortality, Critically ill patients, Dose-response relationship, Restrictive transfusion strategy

## Abstract

**Objectives:**

Plasma transfusion is commonly used in intensive care units, often for non-bleeding indications despite limited evidence. This study aimed to evaluate the association between plasma transfusions and in-hospital mortality among critically ill patients.

**Methods:**

A retrospective cohort study was conducted using data from 282 adult intensive care unit patients who received transfusion therapy between January and December 2020. Patients were grouped by their exposure to plasma transfusion and further by the transfusion volume. First, multivariable Cox regression coupled with propensity score matching was used to control for baseline confounders and quantify the independent association of plasma transfusion with in-hospital mortality. Subsequently, Cox models and Kaplan-Meier survival curves were employed to elucidate the dose-response relationship between transfusion volume and mortality risk, with additional subgroup analyses.

**Results:**

In the unadjusted analysis, plasma transfusion was significantly associated with increased in-hospital mortality (Hazard ratio = 4.199; 95% confidence interval: 2.53–6.97; p-value <0.001). This association persisted after propensity score matching adjusted for key confounders (Hazard ratio = 3.271; 95% confidence interval: 1.30–8.21; p-value = 0.012). Kaplan-Meier survival analysis demonstrated significantly lower survival probabilities in the transfusion group (log-rank p-value <0.05). Furthermore, a dose-dependent relationship was revealed: mortality risk increased at higher volumes, reaching statistical significance at >800 mL (Hazard ratio = 4.09; 95% confidence interval: 1.19–14.04; p-value = 0.025). Subgroup analysis indicated that the elevated risk was particularly pronounced among patients who concurrently received red blood cell transfusions.

**Conclusion:**

Plasma transfusion is independently and dose-dependently associated with increased mortality in critically ill patients, particularly when used without clear indications. These findings support a restrictive, evidence-based approach, emphasizing correction of underlying coagulopathy over prophylactic transfusion. Strict adherence to guidelines and an individualized risk-benefit assessment are essential to improve patient safety.

## Introduction

In the field of critical care medicine, transfusion therapy is frequently employed as a supportive treatment to address anemia, replenish blood volume, and correct coagulopathy [1]. The use of frozen plasma (FP) in intensive care units (ICUs) remains prevalent. Epidemiologic data from a recent large international multicenter study indicate that roughly 10% of critically ill patients receive plasma transfusions during their hospital stay, of which up to 37% are given for non-hemorrhagic indications [2]. This aligns with earlier observations from studies conducted in the UK, Australia, and other countries [3, 4, 5].

Several international guidelines [6, 7, 8, 9, 10] outline limited indications for FP use, such as active bleeding with coagulation factor deficiencies, plasma exchange, specific coagulopathies requiring replacement therapy, and massive transfusion protocols. However, the evidence supporting prophylactic FP use in critically ill patients remains limited and controversial [11]. Studies have shown that early high-ratio plasma transfusion (plasma-to-red-cell ratio ≥1:2) in patients undergoing massive transfusion can reduce mortality rates [12, 13, 14]. Conversely, research conducted in Hiroshima indicated that the lack of improvement in coagulation following FFP transfusion was independently and significantly associated with death within 28 days [15]. Additionally, Qin et al. found that early FFP transfusion in sepsis patients was significantly correlated with an increased risk of mortality [16].

Despite this, plasma transfusion remains prevalent in current clinical practice, with its potential risks often underestimated. A retrospective audit at a tertiary hospital revealed that up to 51.8% of plasma transfusions were administered outside of guideline recommendations, including unnecessary prophylactic use [17]. Such inappropriate transfusions waste limited blood resources and expose patients to a range of transfusion-related adverse effects [18,19].

Employing a retrospective cohort analysis, this study investigates the relationship between plasma transfusion dosage and mortality risk among critically ill patients. It aims to evaluate clinical outcomes to establish evidence-based risk thresholds and provide decision support for optimizing transfusion practices.

## Material and methods

### Study design

This single-center retrospective cohort study was conducted at Hunan Provincial People's Hospital (The First Affiliated Hospital of Hunan Normal University), a tertiary Grade A general hospital directly under the Hunan Provincial Health Commission. As a regional medical center, it holds major responsibilities in clinical care, teaching, research, and the management of critically ill patients. Its comprehensive ICU is equipped with standard monitoring and life-support devices, serving a diverse population of critically ill medical and surgical patients. This setting provided an adequate case source for the study. The admitted cases were patients hospitalized in the ICU who received blood transfusion therapy from January 2020 to December 2020. This study was approved by the hospital’s Ethics Committee.

### Inclusion and exclusion criteria

The inclusion criteria were age ≥18 years and ICU stay ≥24 h. Required core data items were available and complete in all medical records. These data comprised: (1) baseline demographic characteristics (sex, age) and disease severity (Acute Physiology and Chronic Health Evaluation II [APACHE II] score); (2) key pre-transfusion laboratory parameters (hemoglobin [Hb], prothrombin time [PT], activated partial thromboplastin time [APTT], fibrinogen, among others); (3) detailed records of plasma and other blood product transfusions; and (4) in-hospital survival outcomes.

The exclusion criteria were patients aged <18 years, ICU stay <24 h (to minimize the inclusion of patients dying rapidly from their primary disease), and patients receiving plasma exchange. For patients with multiple ICU admissions, data collection was restricted to the hospitalization during which they received their first plasma transfusion.

After applying these criteria, 282 critically ill patients were included in the final cohort.

### Data collection

Plasma transfusion involves the administration of various plasma preparations, including fresh frozen plasma (FFP), frozen plasma (FP), cryoprecipitate-reduced plasma, and cold supernatant. Indications for plasma transfusion are categorized as prophylactic or therapeutic. Prophylactic transfusions correct coagulation abnormalities in non-bleeding patients or reduce perioperative bleeding risks in those undergoing invasive procedures or surgery. Therapeutic transfusions are primarily used for hemostatic support in actively bleeding patients, antithrombin III (AT3) supplementation, and plasma exchange. The indications for plasma transfusion applied in this study were based on contemporary international guidelines [6,9], which align with current clinical practice in China.

Plasma transfusion is indicated in a range of critical clinical scenarios, including severe bleeding due to single or multiple coagulation factor deficiencies; dilutional coagulopathy following massive blood loss or transfusion; cases requiring emergent reversal of warfarin anticoagulation; heparin resistance associated with AT3 deficiency; disseminated intravascular coagulation (DIC); thrombotic thrombocytopenic purpura (TTP) and hemolytic uremic syndrome (HUS); as well as in the setting of major trauma, extensive burns, and large-volume autologous blood salvage (>1000 mL in adults). Plasma transfusion is also utilized during therapeutic plasma exchange procedures.

Patients were divided into two groups, plasma transfusion group and no plasma transfusion group, according to whether they received plasma transfusion during hospitalization in the ICU. The following data were collected: demographic data, ICU hospitalization time, APACHE II score, blood transfusion data, and pre-transfusion laboratory tests, including Hb, C-reactive protein, procalcitonin, PT, APTT, fibrinogen, AT3 activity, total bilirubin, alanine aminotransferase (ALT), creatinine and others.

### Statistical analysis

All analyses were conducted in Statistical Package for Social Sciences (SPSS) 25.0. For continuous variables, normally distributed data are summarized as means ± standard deviation and compared using Student’s *t*-test. Non-normally distributed data are reported as medians and interquartile range and compared using the Wilcoxon test. For categorical variables, data are presented as numbers (percentages) and compared using the Chi-square (χ²) test.

First, multivariable Cox proportional hazards regression was performed on the overall cohort to identify risk factors for mortality and to estimate the unadjusted association between plasma transfusion and patient outcomes, reporting results as hazard ratios (HR) with 95% confidence intervals (95% CI). To better isolate the effect of plasma transfusion by minimizing baseline confounding and enhancing intergroup comparability, 1:1 propensity score matching (PSM) was subsequently performed. In the matched cohort, a Cox proportional hazards model (PSM model) was applied to re-evaluate the association between plasma transfusion and mortality risk. This model was further adjusted for key covariates (age, patient type, PT) to assess robustness. Survival probabilities were visualized using Kaplan-Meier curves, and the log-rank test was used to compare survival between transfusion groups. A two-sided p-value <0.05 was considered statistically significant.

## Results

### Baseline characteristics

A total of 282 critically ill patients met the inclusion criteria, of which 192 survived, accounting for 68.09% of the total number. Baseline characteristics, including gender, age, duration of ICU stay, APACHE II score, and patient category, were similar between the two groups. However, patients in the plasma transfusion group exhibited worse coagulation function (prolonged PT and APTT; lower fibrinogen and AT3 levels), poorer liver function (elevated total bilirubin), and received more platelets and cryoprecipitate. They also had milder anemia (higher Hb levels), received fewer red blood cell (RBC) transfusions, and had a higher in-hospital mortality rate. PSM (1:1) yielded a final cohort of 110 patients for analysis. Comparison of the matched groups demonstrated no statistically significant differences in any baseline characteristics (all p-value >0.05) (Table 1).

**Table 1:** Baseline characteristics after propensity score matching.

Variable	All (n = 110)	Plasma (n = 55)	Non-plasma (n = 55)	Z/χ2	p-value
Sex				0.00^[3]^	1.000
Male	68(61.8%)	34(61.8%)	34(61.8%)		
Female	42(38.2%)	21(38.2%)	21(38.2%)		
Age (years)	58.8 ± 16.1	57.4 ± 15.9	60.1 ± 16.3	0.89^[1]^	0.373
Length of ICU stay (days)	12.3 ± 13.7	12.1 ± 12.7	12.5 ± 14.7	0.49^[2]^	0.625
APACHE Ⅱ score	23.6 ± 7.7	23.4 ± 7.1	23.8 ± 8.3	0.32^[1]^	0.750
Patient category				0.93^[3]^	0.335
Surgical care	63(57.3%)	34(61.8%)	29(52.7%)		
Medical care	47(42.7%)	21(38.2%)	26(47.3%)		
Sepsis				0.69^[3]^	0.405
Yes	15(13.6%)	9(16.4%)	6(10.9%)		
No	95(86.4%)	46(83.6%)	49(89.1%)		
Hemoglobin (g/L)	77.2 ± 26.2	78.5 ± 27.5	75.8 ± 25.0	0.14^[2]^	0.891
C-reactive protein (g/L)	113.0 ± 93.9	116.8 ± 91.4	109.1 ± 97.0	0.65^[2]^	0.515
Procalcitonin (g/L)	17.9 ± 29.6	19.7 ± 30.9	16.0 ± 28.5	0.67^[2]^	0.505
Prothrombin time (s)	14.5 ± 4.3	14.7 ± 5.0	14.2 ± 3.6	0.66^[2]^	0.511
APTT (s)	36.5 ± 16.9	37.0 ± 13.2	36.0 ± 20.1	1.81^[2]^	0.071
Fibrinogen (g/L)	3.9 ± 2.1	4.0 ± 2.1	3.8 ± 2.1	0.61^[2]^	0.544
AT3 (%)	65.8 ± 20.7	66.3 ± 20.9	65.3 ± 20.7	0.14^[2]^	0.888
Total bilirubin (µmol/L)	41.2 ± 54.6	39.7 ± 48.3	42.6 ± 60.6	0.04^[2]^	0.971
ALT (U/L)	220.4 ± 763.9	177.2 ± 491.3	263.6 ± 965.8	0.93^[2]^	0.351
Creatinine (µmol/L)	159.6 ± 162.5	171.0 ± 173.1	148.1 ± 152.0	0.13^[2]^	0.893
RBC transfusion				1.23^[3]^	0.268
Yes	83(75.5%)	39(70.9%)	44(80.0%)		
No	27(24.5%)	16(29.1%)	11(20.0%)		
Platelet transfusion				0.05^[3]^	0.829
Yes	29(26.4%)	14(25.5%)	15(27.3%)		
No	81(73.6%)	41(74.5%)	40(72.7%)		
Cryo transfusion				0.79^[3]^	0.376
Yes	13(11.8%)	5(9.1%)	8(14.5%)		
No	97(88.2%)	50(90.9%)	47(85.5%)		

ICU: Intensive care unit; APTT: activated partial thromboplastin time; ALT: Alanine aminotransferase; AT3: antithrombin III; Cryo: Cryoprecipitate.

Note:^[1]^ = student t-test; ^[2]^ = Wilcoxon test; ^[3]^= Chi-square (χ²) test.

### Risk factors for mortality

The multivariable Cox regression results are presented in Table 2. Significant independent predictors of mortality were age (HR = 1.04 per year increase; p-value = 0.029), plasma transfusion (HR = 3.34; p-value = 0.017), and RBC transfusion (HR = 10.74; p-value = 0.034). Notably, a longer ICU stay was associated with a lower mortality risk (HR = 0.94 per additional day; p-value = 0.043), a result potentially confounded by survivor bias. Furthermore, surgical patient status was a strong protective factor compared to medical patient status (HR = 0.13; p-value = 0.002).

**Table 2:** Predictors of mortality by Cox regression analysis.

Variable	Hazard Ratio	95% CI	p-value
Sex	1.03	0.68–1.56	0.948
Age (years)	1.04	1.03–1.06	0.029*
Patient category	0.13	0.07–0.23	0.002*
Length of ICU stay (days/)	0.94	0.92–0.97	0.043*
APACHE Ⅱ score	1.06	1.03–1.09	0.096
Sepsis	2.52	1.32–4.79	0.231
Hemoglobin (g/L)	1.00	0.98–1.01	0.883
C-reactive protein (g/L)	1.00	1.00–1.00	0.797
Procalcitonin (g/L)	1.01	1.00–1.02	0.408
Prothrombin time (s)	1.10	1.04–1.16	0.130
APTT (s)	1.01	1.00–1.02	0.364
Fibrinogen (g/L)	0.99	0.85–1.15	0.944
AT3 (%)	1.02	1.01–1.03	0.166
Total bilirubin (µmol/L)	1.00	1.00–1.01	0.480
ALT (U/L)	1.00	1.00–1.00	0.464
Creatinine (µmol/L)	1.00	1.00–1.00	0.323
Plasma transfusion	3.34	2.19–5.09	0.017*
RBC transfusion	10.74	4.21–27.39	0.034*
Platelet transfusion	2.16	1.29–3.63	0.214
Cryo transfusion	0.47	0.21–1.05	0.433

95%: CI: 95% Confidence interval; ICU: Intensive care unit; APTT: activated partial thromboplastin time; ALT: Alanine aminotransferase; AT3: antithrombin III; Cryo: Cryoprecipitate.

Cox proportional hazards regression was performed on the propensity score-matched cohort, adjusting for all available covariates.

### Association between plasma transfusion and mortality

As shown in Table 3, plasma transfusion was significantly associated with increased in-hospital mortality. In the unadjusted model (Model 1), the HR was 4.199 (95% CI: 2.53–6.97; p-value <0.001). After adjusting for key covariates (age, patient category, RBC transfusion, and ICU length of stay) via PSM, the association remained significant, albeit attenuated, with an HR of 3.271 (95% CI: 1.30–8.21; p-value = 0.012).

**Table 3:** Association of plasma transfusion with in-hospital mortality.

Model	Hazard Ratio	95% Confidence interval	p-value
Model 1	4.199	2.53–6.97	<0.001*
PSM	3.271	1.30–8.21	0.012*

95%: CI: 95% Confidence interval; PSM: Propensity score-matched.

Model 1 is the univariate Cox regression. The PSM Model is derived from a propensity score-matched analysis, incorporating adjustments for the following covariates: age, patient category, red blood cell transfusion status, and intensive care unit length of stay.

### Subgroup analysis

**Fig. 1:**
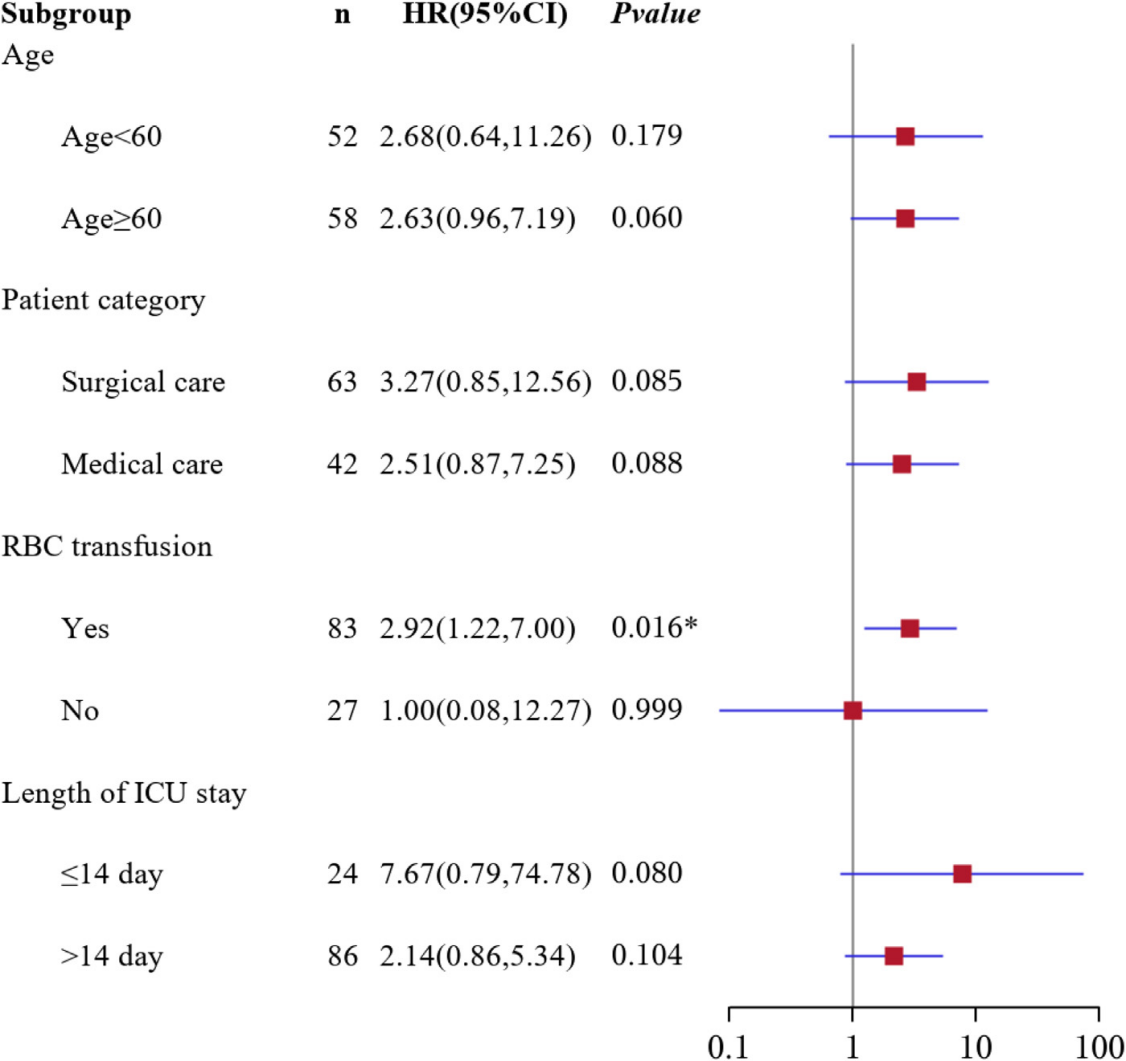
Subgroup analysis of the association between plasma transfusion and in-hospital mortality. The model was fit on the propensity score-matched (PSM) cohort, with adjustment for age, patient category, red blood cell (RBC) transfusion status, and intensive care unit (ICU) length of stay.

Subgroup analyses were performed using the PSM cohort to assess the association between plasma transfusion and mortality risk across strata of age, patient category, RBC transfusion status, and ICU length of stay (Figure 1). After covariate adjustment, a statistically significant association was observed only for the subgroup of patients who had received RBC transfusions (HR = 2.92; 95% CI: 1.22–7.00; p-value = 0.016). For the subgroups stratified by age (<60 or ≥60 years), patient category (surgical or medical), and ICU stay (≤14 or >14 days) the association between plasma transfusion and mortality risk was not statistically significant (all p-value >0.05).

### Dose-response relationship

Following PSM adjustment, a dose-response relationship was observed between plasma transfusion volume and in-hospital mortality (Table 4). Mortality risk increased progressively with higher volumes, reaching statistical significance only at the highest dose. Compared to no transfusion, the risk was elevated but not significant with ≤400 mL (HR = 2.27; 95% CI: 0.86–6.01; p-value = 0.100) and 401–800 mL (HR = 2.52; 95% CI: 0.78–8.10; p-value = 0.121). A significantly higher risk was observed with volumes >800 mL, representing a 4.09-fold increase in mortality (HR = 4.09; 95% CI: 1.19–14.04; p-value = 0.025).

**Table 4:** Association between plasma transfusion volume and in-hospital mortality.

Variable	Hazard Ratio	95% CI	p-value
<400	2.27	0.86–6.01	0.100
401–800	2.52	0.78–8.10	0.121
≥801	4.09	1.19–14.04	0.025*

95%: CI: 95% Confidence interval.

The model was fit on the propensity score-matched cohort, adjusted for age, patient category, red blood cell transfusion status, and intensive care unit length of stay. Patients who received no plasma transfusion (0 mL) served as the reference group.

### Survival analysis

**Fig. 2:**
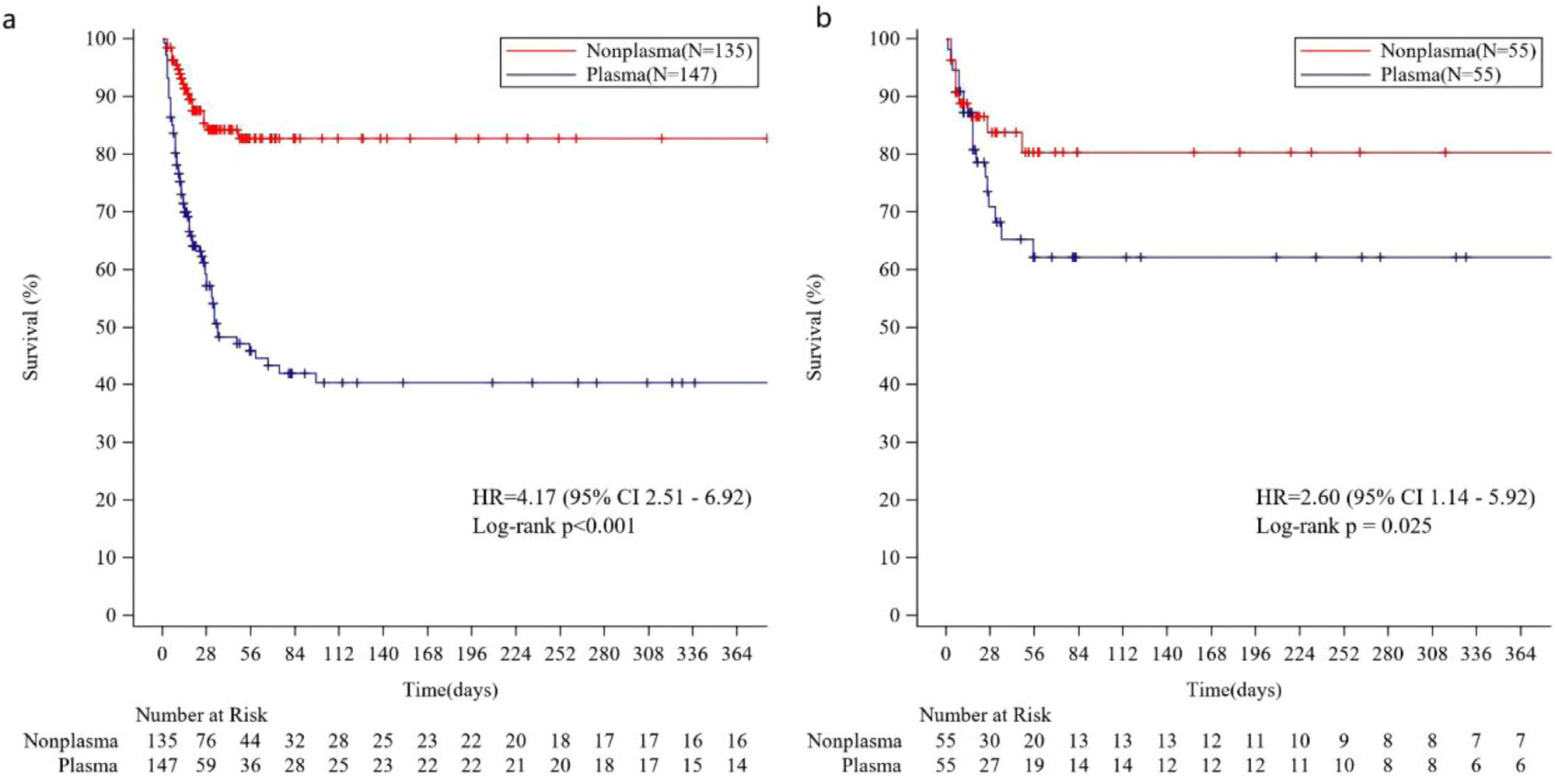
Kaplan-Meier survival estimates. **(**a) Estimates derived from the original (unmatched) cohort. (b) Estimates derived from the propensity score-matched (PSM) cohort.

Survival analysis demonstrated that patients receiving plasma transfusion had significantly lower survival probabilities than non-transfused patients. In the unadjusted analysis, the transfusion group exhibited a 4.17-fold higher mortality risk (Figure 2a). In the PSM cohort, plasma transfusion remained significantly associated with increased mortality, with an adjusted hazard ratio of 2.60 (Figure 2b). The differences in survival curves were statistically significant in both analyses (log-rank p-value <0.05).

## Discussion

This retrospective study of 282 critically ill ICU patients aimed to investigate the impact of plasma transfusion and its dosage on mortality, clarifying the risks and benefits associated with plasma use in critical care. After adjusting for baseline confounders employing PSM, the mortality risk in the plasma transfusion group remained significantly elevated, with a hazard ratio of 3.271 (95% CI: 1.30–8.21; p-value = 0.012). Moreover, a dose-dependent relationship was evident, as the risk increased to 4.09-fold (95% CI: 1.19–14.04; p-value = 0.025) when the transfusion volume exceeded 800 mL.

Patients receiving plasma transfusions presented with poorer baseline clinical characteristics, including more severe coagulopathies, a higher incidence of sepsis, and worse liver function. This suggests that transfusion decisions largely followed clinical indications, targeting sicker patients. However, after balancing 19 baseline variables using PSM (Table 1), plasma transfusion remained independently associated with increased mortality. This indicates that, beyond reflecting illness severity, plasma transfusion itself may confer additional risk.

The increased mortality risk associated with plasma transfusion may be linked to several factors. First, immune-inflammatory storms [20,21], where plasma contains various bioactive substances (complement, cytokines) that can activate systemic inflammatory responses, leading to febrile non-hemolytic transfusion reactions, allergic reactions, transfusion-related acute lung injury (TRALI) [22], and even hemolysis [23], potentially causing direct patient mortality or complicating their condition. TRALI and transfusion-associated circulatory overload (TACO) are leading causes of transfusion-related deaths, identified as independent risk factors for ICU mortality [24,25]. For instance, the UK SHOT report attributed 52.54% of transfusion-related deaths to TACO among a total of 59 cases [26]. Second, although volume overload is effective for volume replacement and coagulation correction [27], critically ill patients often have impaired physiological functions and limited compensatory capacities, making them less tolerant to large-volume plasma transfusions, thus increasing the risk of acute left heart failure, pulmonary edema [28], citrate toxicity-induced hypocalcemia and myocardial depression. Third, plasma administration can exacerbate coagulation imbalance [29]. Because plasma is rich in procoagulant factors such as thrombin and fibrinogen, it may alter blood flow dynamics and disrupt the homeostatic balance, thereby increasing the risk of thrombosis. This can trigger secondary inflammation, tissue edema, or necrosis, ultimately worsening clinical outcomes [30]. A study on FFP transfusion efficacy in ICU patients with decompensated cirrhosis and coagulopathy found no benefit in terms of mortality but increased hospital stays and multi-organ failure risk [31].

Subgroup analysis provided further insights, revealing a statistically significant increase in mortality specifically among patients who received RBC transfusions (HR = 2.92; 95% CI: 1.22–7.00; p-value = 0.016). In contrast, this association was not significant in subgroups stratified by age, patient category, or ICU length of stay. This suggests that patients requiring concomitant RBC and plasma transfusion represent a particularly vulnerable population. In these critically ill patients that are often facing anemia, active bleeding, or hemodynamic instability, plasma may compound risks through volume overload, inflammatory activation, or coagulopathy. Thus, in multi-component transfusion settings, the risks of plasma transfusion appear amplified, necessitating cautious reassessment even when coagulation parameters seem to justify its use. Clinical practice should adhere strictly to evidence-based guidelines, prioritize treating underlying conditions, and exercise heightened vigilance when considering plasma alongside other blood products.

This study has several limitations. First, as a retrospective observational analysis, it may be subject to residual confounding despite adjustment for known factors via PSM. Second, the exact time interval between plasma transfusion and death was not consistently recorded in the medical records, limiting our ability to establish a clear temporal relationship or infer causality. To partially address this, we excluded patients with an ICU stay of <24 h, thereby reducing the likelihood of including deaths unrelated to transfusion or driven solely by the underlying disease. Nevertheless, future studies should aim to collect time-to-event data more systematically to enable time-varying exposure analyses. Third, we were unable to directly quantify specific adverse reactions such as TRALI or TACO, limiting our ability to confirm the proposed mechanisms. Fourth, the sample size was relatively modest, particularly in subgroup and dose-response analyses, which may have reduced statistical power and limited the generalizability of our findings. Therefore, these results should be considered exploratory and warrant validation in larger, multicenter prospective studies.

## Conclusions

This study demonstrates that plasma transfusion is independently associated with significantly increased in-hospital mortality in critically ill patients, with a strong dose-dependent effect. The association remained significant after adjusting for confounding factors in PSM. Plasma use, particularly in the absence of active bleeding or clear indications, may cause more harm than benefit, likely due to complications like TRALI, TACO, and thrombosis.

Current clinical practices frequently diverge from established guidelines, potentially exposing patients to avoidable risks. Consequently, a more restrictive and evidence-based strategy for plasma transfusion is strongly warranted. Clinicians are encouraged to avoid prophylactic use, particularly in hemodynamically stable patients, and should instead focus on addressing the underlying causes of coagulopathies. These findings underscore the need for stricter adherence to transfusion guidelines and highlight the importance of future prospective studies to enhance clinical decision-making in critical care settings.

Plasma transfusion should be regarded as a high-intervention therapy that carries substantial risks and requires meticulous patient-specific evaluation before administration. Future research efforts should aim to refine existing guidelines through well-designed prospective studies and large-scale randomized controlled trials, with the goal of establishing precise, individualized dosing strategies. Ultimately, the objective is to improve patient outcomes by maximizing the therapeutic benefits of plasma transfusion while minimizing unnecessary exposure to risk.

## Funding

This study was supported by the Hunan Provincial Health Commission General Project (No. 202204133544).

## Author contributions

All listed authors have contributed to the manuscript substantially and have agreed to the final submitted version. MZ contributed to the concept of the article, wrote the draft, and modified it. CSL and FY supported in data treatment and analysis and reviewed the article. ZH, SF and FFC helped in data collection and modified the article. XSH reviewed and modified the article. HJG did the data analysis, modified thoroughly the whole article, including the tables and the figures. PL helped in funding and reviewed the article.

## Data availability

The data that support the findings of this study are available from the corresponding author upon reasonable request.

## Conflicts of interest

The authors declare that they have no conflict of interest.
